# Third birth intention of the working women with two existing children in Hainan Island, China: The impact of fertility costs and utility

**DOI:** 10.1016/j.heliyon.2024.e33939

**Published:** 2024-07-02

**Authors:** Lei Qiu, Yingqi Li, Jie Zhong, Xuan Liu, Jindong Ding, Dongxian Zhang

**Affiliations:** a School of Public Health, Hainan Medical University, Hainan, PR China; b School of Management, Hainan Medical University, Hainan, PR China; c Research Unit for General Practice, Department of Public Health, University of Copenhagen, Copenhagen, Denmark; d Research Unit for General Practice, Department of Public Health, University of Southern Denmark, Odense, Denmark

**Keywords:** Birth intention, Working women, Fertility costs, Fertility utility

## Abstract

This study evaluates the impact of fertility costs and utility on the third birth intentions among working women with two children in Hainan, China. A cross-sectional survey was conducted in Hainan Island, China in 2021 using an offline survey. Among 1067 working women with two children, only 8.06 % of participants reported having a third-birth intention. After adjustment for potential confounding factors, higher economic cost (odds ratio = 1.89) and lower succession utility (odds ratio = 5.08) were significantly associated with the lack of intention to have a third child. The analysis further demonstrates that family values significantly modulate these economic and utility considerations, highlighting a strong cultural influence on fertility decisions. This finding underscores the necessity for policies that not only mitigate financial burdens but also promote family values supportive of higher fertility. Such measures are essential for creating a cultural and economic environment conducive to higher birth rates.

## 1 Introduction

China's total fertility rate has reached a historic low of 1.3, as reported by the Seventh Census, highlighting a significant demographic challenge [1]. In response, the “three-child policy” was promulgated in 2021 in the country [2] by the Chinese Government that allowed couples to have up to three children [3]. In light of these policy changes, recent empirical research offers insights into their effects on fertility decisions. Ning et al. (2022) provide an extensive evaluation of the new policy's influence across China, highlighting varied responses based on socio-economic status and cultural norms [4]. Similarly, Chen et al. (2023) focus on the fertility intentions among the childbearing-age population in Central China, illustrating how economic and educational factors play pivotal roles in shaping these decisions [5]. Additionally, Yang et al. (2023) analyze the socio-economic determinants specific to younger women, underscoring how financial stability and higher education levels correlate with reduced fertility intentions [6]. Complementing these findings, Yan et al. (2021) discuss sociodemographic differences across the broader population, providing a detailed look at how various factors such as age and income influence the decision-making process for a third child [7]. Under such a loose fertility policy, the decision to have children has shifted from the national fertility policy level to that of the family. Therefore, women's willingness to have a third child has become the main driver of fertility levels [8]. Fertility intention is a psychological mechanism to express fertility desires and attitudes based on individual or family preference for children. Understanding the fertility intentions of people of childbearing age is essential for successfully implementing the fertility policy.

Fertility intention is a psychological mechanism to express fertility desires and attitudes based on individual or family preference for children [9]. Understanding the fertility intentions of people of childbearing age is essential for successfully implementing the fertility policy. The women of childbearing age who are reluctant to have children often cite the reason they “cannot afford to give birth or raise.” Childbirth is a sociocultural and economic behavior. Fertility intention is influenced by the realities of the situation and the economic weight of costs and utility [10].

Harvey Leibenstein was the first to explain fertility costs and benefits through economics [11]. Fertility is a rational economic behavior by rational economic people to pursue utility maximization starting from “homo economicus”(12). To further strengthen our theoretical foundation based on Leibenstein's work, we integrate insights from recent studies that apply these economic principles to contemporary fertility behaviors. This approach helps us examine how changes in policy and economic conditions influence individual decisions regarding family size within the specific socio-economic context of China's three-child policy. Fertility costs include direct and indirect costs. The direct costs are children's life, education, and entertainment expenses. On the other hand, indirect costs refer to the opportunity costs, such as decreased educational opportunities and working income caused by raising children [13]. Therefore, fertility benefits include hedonic, economic, insurance, and family size expansion utilities [14]. Rational economic people can decide to have a child by weighing the fertility costs, benefits, and economic, social, and cultural factors [15].

Chinese researchers have explored various factors influencing the fertility intentions for a second child in different regions of China [16]. Behind the seemingly free choice lies a combination of factors. Women's fertility intentions are influenced by individual and family factors at the micro level and by policy and cultural factors at the macro level. Studies have been conducted to examine the impact of women's age, education, family income, employment status, family structure, and other factors, yielding mixed results [17]. The factors associated with women's fertility intentions may differ due to sociocultural elements, ethnicity, and region variations. Therefore, it was necessary to conduct further studies to explore factors related to women's fertility intention in China, especially in the context of the “three-child policy.” To the best of our knowledge, existing literature lacks a comprehensive analysis of how fertility costs and utilities impact the fertility intentions of Chinese women. While numerous studies have explored fertility intentions in China, our research uniquely addresses the combined effects of economic and utility factors on the decision to pursue a third child under the new policy framework, providing fresh insights into the socio-economic and cultural dynamics at play in shaping population trends.

For most working women, the golden age of having children is also the best time for career development [18]. These women can afford all or part of the cost of raising a child and have strong self-awareness and the ability to choose as relatively independent individuals in the economy [19]. By examining this group's fertility intentions, the influencing factors of the fertility rate can be identified in the era of the three-child policy. It can also provide a reference for further policy adjustment. Moreover, fertility intention is a subjective potential that may occur or change over time and the life cycle [20]. Only those women who have given birth to two children and are still fertile have the most say in the choice of three children and can directly translate what they want into practical action. Failure to accurately define the survey objects may lead to a significant deviation in the research inference. As the largest island located in the tropical region of China, the environment and culture of Hainan are different from other regions in the Chinese mainland [21]. Hainan's total fertility rate dropped from 1.69 in 2015 to 1.55 in 2020, but it is still significantly higher than the national average of 1.3 [22].

This study critically examines the 2021 ‘three-child policy’ introduced by the Chinese Government, identifying a significant research gap in understanding how economic and utility factors influence third birth intentions among working women in Hainan. Despite policy relaxations, persistently low fertility rates pose a demographic challenge, underscoring the necessity for a targeted study of the policy's effectiveness and the underlying factors at play. We delve into barriers that hinder the policy's effectiveness, such as economic burdens and perceived fertility utilities, which are crucial for understanding how this policy influences the fertility decisions among working women. To gather direct insights, a cross-sectional survey was conducted with working women who have two children on Hainan Island. Our research not only identifies strategies to alleviate fertility concerns among these women but also provides empirical support for enhancing fertility intentions and developing effective measures to complement the three-child policy. Through this analysis, we aim to contribute to the broader demographic strategies needed to address China's pressing population issues.

## 2 Methods

### 2.1 Participants and sampling

Hainan, as the largest island in China, has a unique tropical environment and culture that differ from the mainland. Its total fertility rate, although having dropped from 1.69 in 2015 to 1.55 in 2020, remains significantly higher than the national average of 1.3, making it an ideal location to study the effects of fertility costs and utility on third birth intentions. Haikou, Qionghai, and Sanya on Hainan Island were the research sites. These three cities had large population densities, strong personnel mobility, enterprise clusters, and diverse occupation types, ensuring the diversity of samples. These three cities were randomly chosen from all the cities in Hainan Province using a simple random sampling method. Within each city, one district or county was randomly selected, also using simple random sampling.

The data collection was carried out through an offline survey method. The questionnaire survey was conducted from October 1st to November 1st, 2021. Eligible respondents were located and questionnaires were distributed in densely populated places such as hospitals, shopping malls, and office buildings during weekdays and rest days. The respondents completed the questionnaires on-site. To ensure confidentiality and anonymity, the survey was conducted anonymously, and no personal identifying information was collected. The collected data were stored securely and only accessible to the research team.

The sample size of 1067 was determined using the formula for sample size calculation in prevalence studies:

$$n=\frac{Z^{2}*P\left(1-P\right)}{E^{2}}$$

where *Z* is the *Z*-value (1.96 for a 95 % confidence level), *P* is the estimated prevalence of the outcome (assumed to be 50 % to maximize sample size), and *E* is the margin of error (3 %). This calculation ensures that the sample size is statistically significant and representative of the population. A total of 1100 questionnaires were distributed, of which 1067 were valid, resulting in an effective response rate of 97 %.

For this study's purpose, the subjects should meet the following inclusion criteria:

(i) be at the stage of childbearing age (20–49 years old);
(ii) live in Hainan Province for more than six months;
(iii) working women who have given birth to a second child;
(iv) volunteer to participate in this survey.

## 3 Main outcome

Fertility intentions were measured by the following question: “Are you willing to have a third child?” The response categories were Yes/No/Not sure. Women who answered ‘Yes’ to the question were classified as having third birth intention, and those who answered ‘No’ were categorized as having no third birth intention.

### 3.1 Measurement of fertility costs and utility

To quantitatively assess fertility costs and utility, we employed the Fertility Cost-Utility Questionnaire developed by prominent Chinese scholars [23]. This comprehensive instrument comprises two distinct sub-scales, each tailored to capture nuanced aspects of fertility-related financial and emotional considerations:

1 Fertility Cost Sub-scale:

Economic Cost: Direct financial expenditures related to child-rearing, such as healthcare, education, and daily living expenses.

Opportunity Cost: Indirect costs including lost income and career opportunities due to child-rearing commitments.

Psychological Cost: Emotional and mental health burdens associated with parenting pressures and expectations.

2 Fertility Utility Sub-scale:

Economic Utility: The perceived financial benefits of having children, such as potential future support.

Insurance Utility: Benefits derived from children as a form of social and financial security.

#### 3.1.1 Succession utility: The value associated with passing on family heritage and lineage

Pleasure Utility: The personal satisfaction and joy derived from childbearing and parenthood.

Each item on these sub-scales was rated using a 5-point Likert-type scale, ranging from 1 (strongly disagree) to 5 (strongly agree), where higher scores indicate greater perceived costs or utilities. The validity of the Fertility Cost-Utility Questionnaire was established through confirmatory factor analysis (CFA). The CFA results demonstrated good structural validity with fit indices showing χ2/df = 5.98, CFI = 0.94, TLI = 0.91, and RMSEA = 0.03. Additionally, the internal consistency reliability of the questionnaire was confirmed with Cronbach's alpha values of 0.86 for the overall questionnaire, 0.88 for the fertility cost sub-scale, and 0.81 for the fertility utility sub-scale. These values indicate high internal consistency and reliability of the measurements used in this study."

## 4 Covariates

General information about age, education, occupation type, and monthly household income was gathered by a questionnaire as a baseline. In addition, family characteristics were collected, including the genders of existing children, residence types, and self-assessed marital relationships.

### 4.1 Statistical methods

Descriptive analyses summarized respondents’ characteristics. Categorical data were reported as frequency count and percentages, while numerical variables were reported as mean and standard deviation (SD) or median and Inter-quartile Range (IQR). Rank-sum or chi-square tests were conducted to compare differences in childbearing intentions among different demographic groups, suitable for our categorical data set that did not meet parametric test assumptions. Additionally, the Kruskal-Wallis test was applied to non-normally distributed continuous variables to compare fertility intentions across groups, accommodating data skewness and outliers. Multinomial logistic regression was utilized for exploring the relationships between childbearing intention and fertility costs and utility. This approach is ideal for categorical dependent variables, such as the intention to have a third child, which was categorized as 'Yes', ‘No’, or ‘Unsure’. In our multinomial logistic regression analysis, we have designated ‘Unsure’ as the base category. This decision was informed by our interest in comparing the more definite responses of ‘No’ and 'Yes' against a neutral midpoint, reflecting participants who are undecided about having a third child. Two models investigating potential association were constructed. Model 1 was adjusted for age. Model 2 was additionally adjusted for education, occupation type, monthly income, the genders of existing children, residence type, and self-assessed marital relationship. The results of the logistic regression were presented in odds ratios (ORs) and 95 % confidence intervals (CIs). All comparisons were two-tailed. The significance threshold was set at a *p*-value ≤0.05. Statistical Analysis System (SAS) 9.4 for Windows (SAS Institute Inc., Cary, NC, USA) performed all statistical procedures.

## 5 Results

This study investigated the participant characteristics and their association with third birth intentions (Table 1). The mean age of participants was 35.65 years (SD = 5.41). The majority of respondents were 30 years and above and had a bachelor's degree or above (64.94 %). Specifically, 52.01 % of the respondents had a bachelor's degree, and 12.93 % had a master's degree or above. Only 8.06 % (86 out of 1067) of participants reported having a third birth intention, 13.12 % (140/1067) were unsure, and 78.82 % (841/1067) declared that they had no third birth intention. Further inferential statistical analyses, including chi-square tests and rank-sum tests, assessed the associations between demographic characteristics and third birth intentions. Initial tests indicated that not only education but also monthly household income significantly impacted birth intentions (p < 0.05), as detailed in Table 1. Significant associations were observed between higher education levels and higher monthly household incomes with increased likelihood of third birth intentions (*p* < 0.05), highlighting socioeconomic factors as crucial determinants. The variables, type of occupation, age, residence type, genders of existing children, and self-rated marital relationship, were not associated with the third birth intention (*p* > 0.05).

**Table 1:** Participants’ characteristics and associations with third birth intention(n = 1067).

Variables	Category	Total(n/%)	Third birth intention			*P*	*Test Used*
			Yes(n/%) (n = 86)	Unsure(n/%) (n = 140)	No(n/%) (n = 841)		
Age (years)	Mean ± Standard deviation	35.65 ± 5.41					
	18∼29	114(10.68)	15(13.16)	19(16.67)	80(70.18)	0.175	Rank-sum
	30∼35	370(34.68)	28(7.57)	51(13.78)	291(78.65)		
	36∼40	356(33.36)	26(7.30)	48(33.36)	282(79.21)		
	41+	227(21.27)	17(7.49)	22(9.69)	188(82.82)		
Education	High School and below	179(16.78)	28(15.64)	25(13.97)	126(70.39)	<0.001	Chi-square
	College	195(18.28)	20(10.26)	25(12.82)	150(76.92)		
	Bachelors	555(52.01)	30(5.41)	81(14.59)	444(80.00)		
	Master and above	138(12.93)	8(5.80)	9(6.52)	121(87.68)		
Occupation	civil servant	253(23.71)	19(7.51)	39(15.42)	195(77.08)	0.122	Chi-square
	Public Institution	300(28.30)	25(8.33)	42(14.00)	244(77.67)		
	state-owned enterprise	195(18.26)	15(7.69)	25(12.82)	155(79.49)		
	private enterprise	121(11.34)	11(9.09)	19(15.70)	91(75.21)		
	Freelancers	198(18.37)	20(10.10)	22(11.11)	156(78.79)		
Residential structure	Living with young parents/in-laws	275(25.77)	28(10.18)	42(15.27)	205(74.55)	0.16	Chi-square
	Living with elderly parents/in-laws	317(29.71)	27(8.52)	44(13.88)	246(77.60)		
	Not living with parents/in-laws	475(44.52)	31(6.53)	54(11.37)	390(82.11)		
Monthly household Income	Less than RMB10,000	196(18.37)	16(8.16)	21(10.71)	159(81.12)	<0.005	Rank-sum
	10,001∼20,000RMB	737(69.07)	46(6.24)	103(13.98)	588(79.78)		
	Above RMB20,000	134(12.56)	24(17.91)	16(11.94)	94(70.15)		
Gender	all boys	442(41.42)	31(7.01)	50(11.31)	361(81.67)	0.265	Chi-square
	all girls	347(32.52)	29(8.36)	55(15.85)	263(75.79)		
	Both children	278(26.05)	26(9.35)	35(12.59)	217(78.06)		
Marital relationship	Good	566(53.05)	51(9.01)	81(14.31)	434(76.68)	0.156	Rank-sum
	Moderate	405(37.96)	27(6.67)	53(13.09)	325(80.25)		
	Poor	96(9.00)	8(8.33)	6(6.25)	82(85.42)		

This research also evaluated the distribution of fertility cost and utility of participants with different third birth intentions (Table 2). Participants indicating a third birth intention had median fertility cost scores of 55 (IQR: 45–68), while those with no such intention scored higher, at 60 (IQR: 51–68), suggesting that lower fertility costs are associated with a higher inclination towards expanding the family. The median fertility utility scores were 56.5 (inter-quartile range, 49–63) and 47 (inter-quartile range, 41–52) among those with a third birth intention and those with no third birth intention, respectively. Women with third birth intention were reported to have lower fertility costs (economic and opportunity costs) and higher fertility utility (economic utility, insurance utility, succession utility, and pleasure utility). Due to skewed distributions, the Kruskal-Wallis test showed that the above variables had statistically significant differences among the populations with different third birth intentions, except for the psychological cost (*p* < 0.05).

**Table 2:** Distribution of fertility costs and utility of participants with different third birth intentions.

Variables	Third birth intention			*H*-value	*p*-value
	Yes (n/%) (n = 86)	Unsure (n/%) (n = 140)	No (n/%) (n = 841)		
**Fertility costs**	55 (45,68)	55.5 (46.5,65)	60 (51,68)	15.17	<0.001
Economic	23 [20,28]	24 [21,28]	27 [23,30]	25.65	<0.001
Opportunity	14 [10,19]	14 [10,17]	15 [12,18]	11.50	0.003
Psychological	19 [13,22]	18 (14.5,21)	19 [16,22]	4.10	0.129
**Fertility utility**	56.5 (49,63)	52 (46.5,57)	47 (41,52)	94.98	<0.001
Economic	10 [8,13]	9 [8,11]	9 [6,10]	46.07	<0.001
Insurance	23 [20,27]	21 [18,23]	19 [16,21]	77.05	<0.001
Succession	12 [9,13]	9 [7,11]	7 [5,9]	109.96	<0.001
Pleasure	13 [12,15]	13 [12,15]	12 [11,14]	10.43	<0.005

Note: Values are expressed as median (interquartile range) due to non-normal distribution in the Kruskal-Wallis test.

In our multinomial logistic regression analysis, detailed in Table 3, we adjusted for multiple confounders across two models. Model 1 adjusted for age alone revealed that higher economic costs significantly increased the odds of participants choosing ‘No’ over ‘Unsure’ with an odds ratio of 1.89 (95 % CI: 1.12–3.18). Model 2, which further adjusted for education, occupation type, monthly income, genders of existing children, residence type, and marital satisfaction, provided nuanced insights into how these factors interact to shape fertility decisions. In Model 1, participants with high succession utility (Succession Utility High) demonstrated a clear preference for a third child, evidenced by a lower odds ratio (OR = 0.50) relative to ‘Unsure’, which suggests a positive influence of perceived benefits on fertility decisions. This association remained consistent and was enhanced slightly after adjustment for potential confounding by education, occupation type, monthly income, genders of existing children, residence type, and self-assessed marital relationship. In terms of economic influence, participants facing higher economic costs (Economic Cost) demonstrated an odds ratio (OR) of 1.89, indicating they were 1.89 times more likely to choose ‘No’ over ‘Unsure’. This reflects significant financial barriers against opting for another child. Furthermore, participants reporting lower succession utility (Succession Utility Low) exhibited a strong deterrent effect, with an OR of 5.08, markedly increasing their likelihood of choosing ‘No’ compared to ‘Unsure’. Additionally, those with higher succession utility (Succession Utility High) showed a distinct preference for a third child (‘Yes'), with an odds ratio of 0.50 relative to ‘Unsure’, underlining the positive influence of succession benefits on fertility decisions. These findings underscore the pivotal role that cultural values related to family continuity play in shaping fertility decisions. The strong influence of succession utility suggests that cultural and familial expectations can significantly drive the decision-making process regarding family expansion.

**Table 3:** Multi-variable logistic regression results of fertility cost and utility relationships with third birth intention.

Variables	Model 1		Model 2	
	Third birth intention		Third birth intention	
	Yes	No	Yes	No
Economic cost				
Low	1.00	1.00	1.00	1.00
Medium	0.70 (0.34–1.46)	**1.63 (1.01**–**2.68)**^**a**^	0.71 (0.33–1.52)	**1.89 (1.12**–**3.18)**^**a**^
High	0.57 (0.19–1.67)	1.23 (0.60–2.51)	0.58 (0.19–1.73)	1.41 (0.68–2.95)
Opportunity cost				
Low	1.00	1.00	1.00	1.00
Medium	0.84 (0.40–1.78)	1.11 (0.66–1.84)	1.00 (0.46–2.17)	1.08 (0.65–1.82)
High	1.19 (0.40–3.53)	1.86 (0.88–3.92)	1.26 (0.41–3.83)	1.85 (0.87–3.93)
Psychological cost				
Low	1.00	1.00	1.00	1.00
Medium	0.77 (0.37–1.63)	1.15 (0.70–1.90)	0.77 (0.35–1.68)	1.21 (0.72–2.01)
High	1.28 (0.50–3.29)	1.07 (0.55–2.07)	1.18 (0.44–3.16)	1.18 (0.60–2.29)
Economic utility				
Low	2.80 (0.67–11.67)	1.53 (0.56–4.24)	3.11 (0.70–13.80)	1.38 (0.49–3.89)
Medium	0.73 (0.38–1.37)	1.06 (0.69–1.64)	0.85 (0.44–1.66)	1.07 (0.68–1.68)
High	1.00	1.00	1.00	1.00
Insurance utility				
Low	0.28 (0.07–1.17)	1.61 (0.76–3.43)	0.28 (0.06–1.26)	1.69 (0.78–3.67)
Medium	0.74 (0.38–1.44)	1.38 (0.87–2.17)	0.81 (0.40–1.64)	1.39 (0.87–2.22)
High	1.00	1.00	1.00	1.00
Succession utility				
Low	0.48 (0.13–1.73)	**4.81 (2.34**–**9.88)**^**c**^	1.52 (0.14–1.98)	**5.08 (2.41**–**10.70)**^**c**^
Medium	**0.48 (0.25**–**0.91)**^a^	**1.83 (1.21**–**2.76)**^**b**^	**0.50 (0.26**–**0.97)**^a^	**1.88 (1.23**–**2.87)**^**b**^
High	1.00	1.00	1.00	1.00
Pleasure utility				
Low	1.82 (0.79–4.24)	1.52 (0.83–2.77)	1.49 (0.61–3.61)	1.44 (0.77–2.69)
Medium	0.65 (0.34–1.24)	1.13 (0.73–1.75)	0.59 (0.31–1.15)	1.01 (0.65–1.57)
High	1.00	1.00	1.00	1.00

Note: CI, confidence interval; OR, odds ratio; Model 1: Results were adjusted for age; Model 2: Results were adjusted for education, type of occupation, monthly income, the gender of existing children, type of residence, and self-assessed marital relationship; ^a^:*p* < 0.05; ^b^:*p* < 0.01; ^c^:*p* < 0.0001.

## 6 Discussion

The third-child intention rate of the working women with two existing children in Hainan Island was 8.06 % in this study. This is lower than the 9.4 % reported in Zhu's study of participants with two children in Shanghai, China [24], and lower than the 12.2 % reported by Yan in 2021 [25]. Our study's focus on working women with two existing children controlled for confounding factors, providing more detailed and accurate insights. The third-child intention among these women is often influenced by rational considerations related to career development and the experience of raising two children [26]. This intention is generally lower than that of the general female population in China [27]. Our study also found that 13.12 % of working women were uncertain about having a third child, highlighting a potential target group for policies aimed at increasing third-child birth rates. Measures should be designed to support these women in making informed decisions about having three children.

This study elucidated the effects of fertility cost and utility on working women's willingness to have a third child. Our multi-factor analysis found that higher economic costs negatively impacted third birth intention. As the cost of child education continues to rise, financial considerations become a major factor in family planning decisions [28]. Economic costs, including child-rearing and education expenses, deter many families from having three children due to the significant financial strain. The China Health and Family Planning Commission reported in 2017 that childbearing expenses constitute nearly half of many families' incomes [29]. High economic expenditure on fertility affects the life quality of families [30]. If the economic cost of raising three children is added, it will put heavy economic pressure on many families and force them to choose to give up the plan of having three children.

In addition, this study found a stable effect of succession utility on occupational female fertility intentions. According to the adjusted ORs for succession utility, the succession utility positively affected the third birth intention of the working women with two existing children. The working women with high succession utility were 5.08 times more likely to have a third birth intention than those with low succession utility levels. This finding contrasts with previous research, which suggested that the traditional Chinese fertility concept of ‘the more children, the more blessings' has diminished among urban youth [31]. However, geographical and cultural differences might explain this inconsistency. Hainan Province, located in China's southernmost region, differs socio-economically and culturally from the mainland. In 2020, Hainan's birth rate was 10.36 per thousand, compared to the national average of 8.52 per thousand [32]. Hainan's culture still values multiple births, and the traditional belief in having more children for blessings remains prevalent. Fertility and the birth of children are highly valued, and women are seen as key to continuing family traditions. The practice of ‘carrying on the family line’ through procreation is deeply rooted in Hainanese culture. [33], explaining the significant impact of succession utility on third birth intentions.

This study is the first to focus on the third-child intention among working women with two existing children in China. However, the sample was confined to working women in Hainan Island, limiting the generalizability of the findings to the entire Chinese population. Future studies should include more representative samples from different regions in China to better understand third birth intentions among working women.

## 7 Conclusion

The third-child intention rate of the working women with two existing children in Hainan Island was 8.06 % in this study. The impact of economic cost and succession utility on the third birth intention of the working women in Hainan Island was significant. Based on the results of this study, the following suggestions were proposed. It is necessary to transfer or share the economic costs of child-rearing for people of childbearing age at the national level, such as providing second-child and mortgage subsidies. On the other hand, young people's conception of marriage, childbearing, strengthening family, and responsibility should be gradually changed. The state should continue strengthening family construction and promoting the “family” culture. Most young people emphasize individualism excessively under the influence of various factors. In terms of policy and public messaging, it is essential to place greater emphasis on promoting family values and responsibilities, and to actively create a cultural environment that supports reproductive decisions. By strengthening the narrative around the importance of family lineage and the joys of larger families, we can cultivate a societal context that not only supports but encourages decisions to expand families. This approach aligns with our findings on succession utility and could effectively enhance third birth intentions among the population.

## Ethics approval and consent to participate

This study was conducted in accordance with the principles outlined in the Declaration of Helsinki and was approved by the Human Research Ethics Committee, Hainan Medical University, Haikou, China (HYLL-2022-210). After the study procedures were explained to the participants, written informed consent was obtained from them in accordance with the Declaration of Helsinki.

## Data availability statement

Data associated with this study are not publicly available due to privacy and ethical considerations. The data involve sensitive personal information collected from women in Hainan Province under the condition of confidentiality as assured to the participants prior to data collection. Disclosure of this data could compromise the privacy of the individuals involved. As such, the dataset is maintained under strict confidentiality protocols to ensure participant privacy and is available only under conditions that comply with these ethical considerations. Requests for access to the data can be directed to the corresponding author. The corresponding author will consider such requests in accordance with ethical guidelines and existing confidentiality agreements.

## CRediT authorship contribution statement

**Lei Qiu:** Writing – original draft. **Yingqi Li:** Methodology, Investigation. **Jie Zhong:** Writing – original draft. **Xuan Liu:** Writing – original draft. **Jindong Ding:** Writing – review & editing, Conceptualization. **Dongxian Zhang:** Writing – original draft, Conceptualization.

## Declaration of competing interest

The authors declare that they have no known competing financial interests or personal relationships that could have appeared to influence the work reported in this paper.

## References

[1] Akimov A.V., Gemueva K.A., Semenova N.K. (2021). The Seventh population Census in the PRC: results and prospects of the country's demographic development. Herald Russ. Acad. Sci..

[2] The State Council of The People’s Republic of China.Third-Child Policy to Unleash Childbirth Potential.http://english-www-gov-cn-swebvpnhainmceducn:8118/statecouncil/ministries/202107/22/content_WS60f8c05ac6d0df57f98dd5efhtml.

[3] Tatum M. (2021). China's three-child policy. Lancet (London, England).

[4] Ning N., Tang J., Huang Y., Tan X., Lin Q., Sun M. (2022). Fertility intention to have a third child in China following the three-child policy: a cross-sectional study. Int. J. Environ. Res. Publ. Health.

[5] Chen Q., Wang A., Song X., Liu X., Liu Y., Wei J., Shu J., Sun M., Zhong T., Luo M., Wang T., Zhang S., Xie D., Qin J. (2023). Fertility intentions to have a second or third child among the childbearing-age population in Central China under China's three-child policy: a cross-sectional study. J Glob Health.

[6] Yang H., Han R., Wang Z. (2023). Third-child fertility intention and its socioeconomic factors among women aged 20-34 years in China. BMC Publ. Health.

[7] Yan Z., Hui L., Wenbin J., Liuxue L., Yuemei L., Bohan L., Lili W. (2021). Third birth intention of the childbearing-age population in mainland China and sociodemographic differences: a cross-sectional survey. BMC Publ. Health.

[8] Schwartz S.R., Baral S. (2015). Fertility-related research needs among women at the margins. Reprod. Health Matters.

[9] M N.B., Beaujouan E., Berrington A. (2010). Stability and change in fertility intentions in Britain, 1991-2007. Popul. Trends.

[10] Bachrach C.A., Morgan S.P. (2013). A cognitive-social model of fertility intentions. Popul. Dev. Rev..

[11] Wu A.K., Elliott P., Katz P.P., Smith J.F. (2013). Time costs of fertility care: the hidden hardship of building a family. Fertil. Steril..

[12] M N.B., Beaujouan E. (2012). Fertility postponement is largely due to rising educational enrolment. Popul. Stud..

[13] Testa M.R. (2014). On the positive correlation between education and fertility intentions in Europe: individual- and country-level evidence. Adv. Life Course Res..

[14] Qian Y., Liu X.Y., Fang B., Zhang F., Gao R. (2020). Investigating fertility intentions for a second child in contemporary China based on user-generated content. Int. J. Environ. Res. Publ. Health.

[15] Liu J., Zhang L. (2021). Fertility intention-based birth forecasting in the context of China's universal two-child policy: an algorithm and empirical study in Xi'an City. J. Biosoc. Sci..

[16] Zheng Y., Yuan J., Xu T., Chen M., Liang H., Connor D. (2016). Socioeconomic status and fertility intentions among Chinese women with one child. Hum. Fertil..

[17] Jiang Q., Li Y., Sanchez-Barricarte J.J. (2016). Fertility intention, son preference, and second Childbirth: survey findings from shaanxi Province of China. Soc. Indicat. Res..

[18] Zhang L., Liu J., Lummaa V. (2022). Intention to have a second child, family support and actual fertility behavior in current China: an evolutionary perspective. Am. J. Hum. Biol. : the official journal of the Human Biology Council.

[19] Zhang X., Huang J., Luo Y. (2021). The effect of the universal two-child policy on medical insurance funds with a rapidly ageing population: evidence from China's urban and rural residents' medical insurance. BMC Publ. Health.

[20] Wang T., Wang C., Zhou Y., Zhou W., Luo Y. (2019). Fertility intentions for a second child among urban working women with one child in Hunan Province, China: a cross-sectional study. Publ. Health.

[21] Rondinelli C., Lin T.-S., Billari F. (2010). Women's wages and childbearing decisions: evidence from Italy. Demogr. Res..

[22] Kim Y.Y., Kang H.J., Ha S., Park J.H. (2019). Effects of living in the same region as one's workplace on the total fertility rate of working women in Korea. Epidemiology and health.

[23] Tan X. (2015). The factors influencing the fertility intention for second child under China's new fertility policy in the perspective of cost-utility theory:an analysis of the survey data in XuZhou. South China Popul..

[24] Zhu C., Yan L., Wang Y., Ji S., Zhang Y., Zhang J. (2022). Fertility intention and related factors for having a second or third child among childbearing couples in Shanghai, China. Front. Public Health.

[25] Yan Z., Hui L., Wenbin J., Liuxue L., Yuemei L., Bohan L. (2021). Third birth intention of the childbearing-age population in mainland China and sociodemographic differences: a cross-sectional survey. BMC Publ. Health.

[26] Debelew G.T., Habte M.B. (2021). Contraceptive method utilization and determinant factors among young women (15-24) in Ethiopia: a mixed-effects multilevel logistic regression analysis of the performance monitoring for action 2018 household survey. BioMed Res. Int..

[27] Hamm M., Miller E., Jackson Foster L., Browne M., Borrero S. (2018). "The financial is the main issue, it's not even the child": exploring the role of finances in men's concepts of fatherhood and fertility intention. Am. J. Men's Health.

[28] Xiang Z., Zhang X., Li Y., Li J., Wang Y., Wang Y., Ming W.K., Sun X., Jiang B., Zhai G., Wu Y., Wu J. (2023). Fertility intention and its affecting factors in China: a national cross-sectional survey. Heliyon.

[29] Zhang S., Ju H. (2021). The regional differences and influencing factors of tourism development on Hainan Island, China. PLoS One.

[30] Marois G., Gietel-Basten S., Lutz W. (2021). China's low fertility may not hinder future prosperity. Proc. Natl. Acad. Sci. U.S.A..

[31] Yu X., Liang J. (2022). Social norms and fertility intentions: evidence from China. Front. Psychol..

[32] Li H.T., Tang J.L., Qiao J. (2024). China's declining fertility rate. BMJ.

[33] Sukontamarn P., Asadullah M.N., Photphisutthiphong N., Nguyen Y.T.H. (2023). Happiness in old age: the daughter connection. J. Happiness Stud..

