# Supplementary material for: Third birth intention of the working women with two existing children in Hainan Island, China: The impact of fertility costs and utility
Source: Heliyon. 2024 Jul 2;10(13):e33939. doi: 10.1016/j.heliyon.2024.e33939 (PMC11282997; doi:10.1016/j.heliyon.2024.e33939)
Supplement: Multimedia component 1 [file mmc1.doc]

**海南省育龄期女性生育意愿调查**

尊敬的女士：

您好，非常感谢您参加此次海南省自然科学基金项目调查组关于生育意愿现状的调查，祝您身体健康！本问卷实行匿名制，所有数据仅用于学术研究，不会给您带来任何不良影响和后果，请您根据您的实际情况放心填写！再次感谢您的参与！

1. 您的年龄（周岁）：

2.您的民族：

○汉族

○黎族

○苗族

○其它少数民族

3.您的文化程度：

○初中及以下

○高中或中专

○高职或大专

○大学本科

○硕士及以上

1. 您现有孩子数量？

○目前没有孩子

○1个孩子

○2个孩子

○3个孩子及以上

5.您目前现有孩子的性别：

- 目前没有孩子
- 只有男孩
- 只有女孩
- 儿女双全

6.您认为一般家庭有几个孩子最理想？

- 1个孩子
- 2个孩子
- 3个孩子
- 4个孩子及以上

7.您打算生育几个孩子？（根据实际情况对个人生育子女数量的打算）

- 1个孩子
- 2个孩子
- 3个孩子
- 4个孩子及以上

8.再要一个孩子对您来说是否重要？

- 不重要
- 不太重要
- 有点重要
- 非常重要

| 9.孩子抚养成本很高，给家庭经济带来很大压力 | 完全赞同 比较赞同 不确定 不太赞同 非常不赞同 |
| --- | --- |
| 10.孩子教育成本很高，给家庭经济带来很大压力 | 完全赞同 比较赞同 不确定 不太赞同 非常不赞同 |
| 11.孩子越多将会降低家庭生活质量 | 完全赞同 比较赞同 不确定 不太赞同 非常不赞同 |
| 12.孩子越多将会降低孩子教育质量 | 完全赞同 比较赞同 不确定 不太赞同 非常不赞同 |
| 13.物价太高，家人怕生活质量下降，不愿生育二孩 | 完全赞同 比较赞同 不确定 不太赞同 非常不赞同 |
| 14.孩子教育成本太高，家人认为在现有经济条件下只适合生育一个孩子 | 完全赞同 比较赞同 不确定 不太赞同 非常不赞同 |
| 15.生育孩子导致自己（或配偶）放弃受教育机会 | 完全赞同 比较赞同 不确定 不太赞同 非常不赞同 |
| 16.生育抚养孩子导致自己（或配偶）丧失工作晋升机会 | 完全赞同 比较赞同 不确定 不太赞同 非常不赞同 |
| 17.生育抚养孩子耽误自己（或配偶）大量工作时间，导致家庭收入减少 | 完全赞同 比较赞同 不确定 不太赞同 非常不赞同 |
| 18.工作压力大，不能放弃工作生育小孩 | 完全赞同 比较赞同 不确定 不太赞同 非常不赞同 |
| 19.生育抚养孩子太麻烦，使自己 （或配偶） 丧失大量自由时间，无法做自己喜欢的事 | 完全赞同 比较赞同 不确定 不太赞同 非常不赞同 |
| 20.生育抚养孩子太累，找不到合适的人帮忙带孩子 | 完全赞同 比较赞同 不确定 不太赞同 非常不赞同 |
| 21.总是担心孩子教育质量，害怕自己的孩子不如别人的孩子，心理压力很大。 | 完全赞同 比较赞同 不确定 不太赞同 非常不赞同 |
| 22.担心与孩子不能很好地沟通，相处困难。 | 完全赞同 比较赞同 不确定 不太赞同 非常不赞同 |
| 23.担心第二个孩子与第一个孩子相互嫉妒， 争风吃醋，对孩子造成伤害。 | 完全赞同 比较赞同 不确定 不太赞同 非常不赞同 |

| 24.孩子未来工作后可以增加家庭财富 | 完全赞同 比较赞同 不确定 不太赞同 非常不赞同 |
| --- | --- |
| 25.孩子可以为父母未来的养老提供经济保障 | 完全赞同 比较赞同 不确定 不太赞同 非常不赞同 |
| 26.多个孩子可以分担未来的养老压力 | 完全赞同 比较赞同 不确定 不太赞同 非常不赞同 |
| 27.孩子未来可以照顾父母 | 完全赞同 比较赞同 不确定 不太赞同 非常不赞同 |
| 28.多个孩子可以减少空巢的风险 | 完全赞同 比较赞同 不确定 不太赞同 非常不赞同 |
| 29.多个孩子可以相互帮助和促进，减少孩子教育失败的风险 | 完全赞同 比较赞同 不确定 不太赞同 非常不赞同 |
| 30.多个孩子会避免太溺爱一个孩子导致其自私自利的性格缺失， 有利于孩子的成长 | 完全赞同 比较赞同 不确定 不太赞同 非常不赞同 |
| 31.孩子越多未来父母养老更有保障 | 完全赞同 比较赞同 不确定 不太赞同 非常不赞同 |
| 32.多个孩子可以避免一个孩子太孤单，太孤僻 | 完全赞同 比较赞同 不确定 不太赞同 非常不赞同 |
| 33.家族兴旺对自己（或配偶）很重要，并且孩子越多家族越兴旺 | 完全赞同 比较赞同 不确定 不太赞同 非常不赞同 |
| 34.第一胎是女孩，希望能生个男孩传宗接代 | 完全赞同 比较赞同 不确定 不太赞同 非常不赞同 |
| 35.生儿育女就是为了延续香火，并且对自己 （或配偶）来说很重要 | 完全赞同 比较赞同 不确定 不太赞同 非常不赞同 |
| 36.自己（或配偶）很喜欢小孩 | 完全赞同 比较赞同 不确定 不太赞同 非常不赞同 |
| 37.孩子使家人情感上更能得到满足 | 完全赞同 比较赞同 不确定 不太赞同 非常不赞同 |
| 38.孩子成长过程中给自己及家人带来很多快乐 | 完全赞同 比较赞同 不确定 不太赞同 非常不赞同 |

第三部分

1. 您第一个孩子的性别：

**男孩**

**女孩**

**儿女双全**

**40.**目前夫妻关系如何？

○非常好

○比较好

○一般

○比较差

○非常差

41.目前婆媳关系如何？

○非常好

○比较好

○一般

○比较差

○非常差

42.您是否打算再要一个孩子？

- 肯定会要
- 可能会要
- 没想好
- 可能不要
- 肯定不要

1. 您认为自己家里的经济条件如何：

○非常好

○比较好

○一般

○比较差

○非常差

44.您认为最近半年自己的健康状况如何：

○非常好

○比较好

○一般

○比较差

○非常差

45.您目前的居住安排是：

- 与年轻父母/岳父母同住（年龄65岁以上）
- 与年老的父母/岳父母同住（年龄65岁以下）
- 不予父母/岳父母同住

46.您在工作日（周一到周五）花在家务劳动时间（小时）：

- 不做家务
- 1小时以下
- 1-2小时
- 3-4小时
- 4小时以上

47.您丈夫在工作日（周一到周五）花在家务劳动时间（小时）：

- 不做家务
- 1小时以下
- 1-2小时
- 3-4小时
- 4小时以上

48.您和您丈夫是否是独生子女？

○夫妻有一方是独生子女

○夫妻都是独生子女

○夫妻都不是独生子女

49.您的现居地；

○城镇

○乡村

50.您的职业是：

○无工作

○务农

○机关事业单位

○企业员工

○教师

○医务工作者

○个体户

○农民工

○其他

1. 我家庭经济上负担得起(再)生育一个孩子

完全赞同

比较赞同

不确定

不太赞同

非常不赞同

1. 家人能帮忙照看，我可以(再)生育一个孩子

完全赞同

比较赞同

不确定

不太赞同

非常不赞同

1. 我家老人认为应该(再)生育一个孩子

完全赞同

比较赞同

不确定

不太赞同

非常不赞同

1. 我的配偶认为应该(再)生育一个孩子

完全赞同

比较赞同

不确定

不太赞同

非常不赞同

| 55.当遇到问题时，你可以从家人那里得到满意的帮助 | ①经常这样 ②有时这样 ③很少这样 |
| --- | --- |
| 56.你很满意与家人讨论各种事情及分担问题的方式 | ①经常这样 ②有时这样 ③很少这样 |
| 57.当你想做一件事情时，家人都能接受且予以支持 | ①经常这样 ②有时这样 ③很少这样 |
| 58.你很满意家人对你表示关心和爱护的方式 | ①经常这样 ②有时这样 ③很少这样 |
| 59.你很满意现在与家人生活的方式 | ①经常这样 ②有时这样 ③很少这样 |
